# Homocysteine levels correlate with velocimetric parameters in patients with erectile dysfunction undergoing penile duplex ultrasound

**DOI:** 10.1111/andr.13169

**Published:** 2022-03-08

**Authors:** Gianmaria Salvio, Alessandro Ciarloni, Simone Cordoni, Melissa Cutini, Nicola Delli Muti, Federica Finocchi, Francesca Firmani, Lara Giovannini, Michele Perrone, Giancarlo Balercia

**Affiliations:** ^1^ Division of Endocrinology Department of Clinical and Molecular Sciences Polytechnic University of Marche Ospedali Riuniti Ancona Italy

**Keywords:** cardiovascular disease, homocysteine, sexual dysfunction

## Abstract

**Introduction:**

Hyperhomocysteinemia may contribute to the development of endothelial dysfunction and, consequently, atherosclerosis, a systemic disease involving the vessels that may affect the cavernous arteries leading to vasculogenic erectile dysfunction. Our study aims therefore to explore the relationship between homocysteine levels and velocimetric parameters detected by basal penile duplex ultrasound such as peak systolic velocity and flaccid penile acceleration in patients with erectile dysfunction.

**Methods:**

A cross‐sectional study was conducted collecting clinical, metabolic, hormonal, and instrumental (basal penile duplex ultrasound) data in patients affected by vasculogenic erectile dysfunction.

**Results:**

Data of 126 subjects affected by erectile dysfunction were collected. Mean age was 52.1 ± 12.6 years, whereas mean body mass index was 25.6 ± 4.0 kg/m^2^. Basal penile duplex ultrasound showed peak systolic velocity values of 13.1 ± 2.9 cm/s and mean flaccid penile acceleration of 2.28 ± 0.70 m/s^2^, with a strong correlation among these two parameters (*r* = 0.690; *p* < 0.001). Frankly pathological values of peak systolic velocity and flaccid penile acceleration were detected in 39.7% and 4.8% of the subjects examined, respectively. Mean homocysteine levels were 14.9 ± 9.5 μmol/l. Homocysteine values >15 μmol/l were found in 26% of the subjects with erectile dysfunction. Peak systolic velocity values and homocysteine levels showed an inverse correlation (*r* = –0.213; *p* = 0.03). Similarly, flaccid penile acceleration values were inversely correlated to homocysteine levels (*r* = –0.199; *p* = 0.05). In addition, an inverse correlation was found between both peak systolic velocity and flaccid penile acceleration and body mass index, atherogenic lipid pattern, and age. Homocysteine and metabolic parameters showed no significant correlations.

**Conclusion:**

Hyperhomocysteinemia is highly prevalent in erectile dysfunction patients. The results of our study show that homocysteine levels correlate with velocimetric parameters assessed by basal penile duplex ultrasound, confirming the role of hyperhomocysteinemia in the genesis of erectile dysfunction of arterial origin.

## 1 INTRODUCTION

Erectile dysfunction (ED), defined as the persistent inability to obtain or maintain penile erection sufficient for a satisfactory sexual performance,^1^ is a common condition in middle aged men. According to data from the Massachusetts Male Aging Study (MMAS), its prevalence increases with aging, affecting up to half of men aged 40–70 years and reaching 70% in older men.^2^ The etiology of ED is multifactorial, involving vasculogenic, neurogenic, hormonal, and pharmacological causes, but impaired penile blood flow is the leading mechanism.^1^ Normal erectile function depends on the release of nitric oxide (NO) in the penile tissue by the endothelium and neurons following the stimulation of non‐adrenergic, non‐cholinergic nerves. In the corpora cavernosa, NO binds to soluble guanylin cyclase, stimulating the production of 3′‐5′‐cyclic guanosine monophosphate (cGMP). The latter activates protein kinase G (PKG) to form a complex cGMP/PKG which induces the relaxation of smooth muscle with consequent hyper‐flow of arterial blood and the onset of penile tumescence.^3^ One of the main mechanisms underlying vascular ED is the endothelial dysfunction, which is accompanied by impaired expression and activation of NO synthase (NOS).^4^ Endothelial dysfunction represents the first step in the origin of atherosclerosis^5^ and underlies the development of cardiovascular disease (CVD), explaining, at least in part, the close link between the latter and ED. Indeed, about the 20% of ED patients may have asymptomatic CVD and the extent of the latter strongly correlates with the severity of ED.^2^ Recently, clinical and experimental findings have shown that hyperhomocysteinemia (HHcys) can alter the homeostasis of the endothelium and contribute to the development of atherosclerosis.^6^ Homocysteine (Hcys) is a sulfur‐containing amino acid deriving from excess dietary methionine though a process called “trans‐sulfuration.” On the opposite, Hcys can be re‐methylated to methionine by the enzymes methionine‐synthase and 5,10‐methylenetetrahydofolate reductase (MTHFR), with vitamin B12 or folic acid (FA) as a cofactor, respectively. When the re‐methylation pathway of Hcys is defective, because of congenital (e.g., genetic defects of MTHFR) or acquired conditions (including vitamins B6 and B12 and FA deficiencies and renal impairment), HHcys may occur.^7^ To explain the relationship between CVD and HHcys, three different pathophysiological mechanisms have been proposed: (1) the reduction of the bioavailability of NO at endothelial level, (2) the direct cytotoxic effect of Hcys mediated by oxidative stress, and (3) dysregulation of the folate metabolism.^8^ According to a recent meta‐analysis, HHcys is more often observed in subjects with ED rather than controls, with a standardized mean difference of 1.00 (95% confidence interval (CI) 0.65–1.35, *p* < 0.0001).^9^ Moreover, MTHFR polymorphisms with lower enzyme activity (namely C677TT and A1298C) are associated to a threefold increased risk for ED.^10^

Dynamic penile duplex ultrasound (D‐PDU) classically represents the gold standard for the diagnostics of men with vascular ED.^11^ After intracavernous injection of a vasoactive drug (e.g., 10 μg alprostadil), color doppler recording of both the cavernous arteries is performed: a peak systolic velocity (PSV) of 35 cm/s or greater indicates arterial sufficiency, whereas a PSV < 25 cm/s is considered diagnostic of arterial insufficiency.^12^ In the last 20 years, duplex ultrasound evaluation of cavernosal PSV in the penile flaccid state has emerged as an alternative, faster, cheaper, and safer technique.^13^ Indeed, a basal‐PSV <13 cm/s is predictive for dynamic‐PSV <25 and <35 cm/s with an accuracy of 89% and 82%, respectively,^14^ without the possible side effects of intracavernous injection of the vasoactive drugs. In addition, the evaluation of flaccid penile acceleration (FPA), calculated using PSV, end diastolic velocity (EDV), and systolic rise time (SRT) according to the formula (PSV ‐ EDV)/SRT, allows to identify subjects with high risk for CVD, because an FPA <1.17 m/s^2^ is associated to a threefold increase of major adverse cardiovascular events.^15^

Thus, the present study aims to evaluate the correlation between basal penile duplex ultrasound (B‐PDU) data as objective indexes of endothelial function, cardiovascular risk factors, and circulating levels of homocysteine Hcys in patients affected by ED.

## 2 MATERIAL AND METHODS

### 2.1 Study design and participants

We enrolled 126 consecutive andrological outpatients affected by ED from the Endocrinology Clinic, Clinic and Molecular Sciences Department, University Hospital “Ospedali Riuniti” of Ancona, Italy, from April 2015 to January 2020. Subjects were included according to the following criteria: any sexual activity in the last 6 months; International Index of Erectile Dysfunction (IIEF‐5) ≤21; ability to understand and provide informed consent. The exclusion criteria were hormonal diseases or uncontrolled diabetes mellitus, recent cardiovascular or cerebrovascular accidents, hormone therapy, and assumption of antineoplastic drugs or of any drug with well‐known negative effects on erectile function (e.g., β‐blockers, antidepressants). All participants provided written informed consent to take part to the study. The study protocol was approved by the local ethics committee (number of protocol 2017‐0222 OR).

### 2.2 Outcome measures

All patients underwent full physical examination, including blood pressure, weight, and height measurement. The following biochemical parameters were considered: follicle‐stimulating hormone (FSH), luteinizing hormone (LH), prolactin, total testosterone, sexual hormone‐binding globulin (SHBG), glucose, insulin, glycosylated hemoglobin (HbA1c), total cholesterol, low‐density lipoprotein (LDL) cholesterol, high‐density lipoprotein (HDL) cholesterol, triglycerides, Hcys, and prostate specific antigen (PSA). Blood samples were taken in the morning, and their assay was carried out as previously specified.^16^, ^17^, ^18^ Normal ranges for adults were 1.4–16.0 IU/L (FSH), 1.3–9.0 IU/L (LH), 2.0–17.0 ng/ml (prolactin), 14.5–48.4 nmol/L (SHBG), 2.41–8.27 ng/ml (total testosterone), 3.4–5.4 g/dl (albumin), 70.0–130.0 mg/dl (glycemia), <25.0 μIU/ml (insulin), <42.0 mmol/mol (HbA1c), <200.0 mg/dl (total cholesterol), <100.0 mg/dl (LDL), 40.0–80.0 mg/dl (HDL), <150.0 mg/dl (triglycerides), and ≤4.0 ng/ml (PSA). Hcys concentration was determined by chemiluminescent microparticle immunoassay (CMIA). According to the normal range of Hcys (5–15 μmol/L), HHcys was set at plasma levels >15 μmol/L. The homeostatic model assessment—insulin resistance (HOMA‐IR) index was also calculated according to the formula (glycemia × insulin)/405: normal range 0.22–2.5 (HOMA‐IR index).

All patients underwent B‐PDU examination (HD 7XE Philips, Bothell, WA, USA) with a 5–12 MHz linear transducer in both the B‐mode and the color‐flow mode. After transversal B‐mode investigation of any penile abnormalities, color doppler recording of the cavernous arteries was performed at the peno‐scrotal junction along an arterial segment corresponding to a Doppler angle of 55°–65°, as described by Aversa and Sarteschi^12^ A PSV <13 cm/s was considered suggestive for arterial ED, as suggested by Corona et al.^14^ Moreover, FPA <1.17 m/s^2^ was considered abnormal according to Rastrelli et al.^15^

### 2.3 Statistical analysis

Statistical analyses were performed using the Statistical Package for Social Sciences (spss) version 22.0 (SPSS Inc., Chicago, IL, USA) for Microsoft Windows. Normal distribution for continuous variables was assessed using the Shapiro–Wilk test for normality if not evident from the histogram and/or normality graph. Data are shown as mean ±  SD. Comparison between groups was performed by *T*‐test for independent samples in case of normality, and by Mann–Whitney *U*‐test in case of non‐normal distributions. Bivariate correlations were investigated by Pearson's test or Spearman's test (whether the distribution was normal or not). Values of *p* < 0.05 were considered statistically significant.

## 3 RESULTS

**FIGURE 1:**
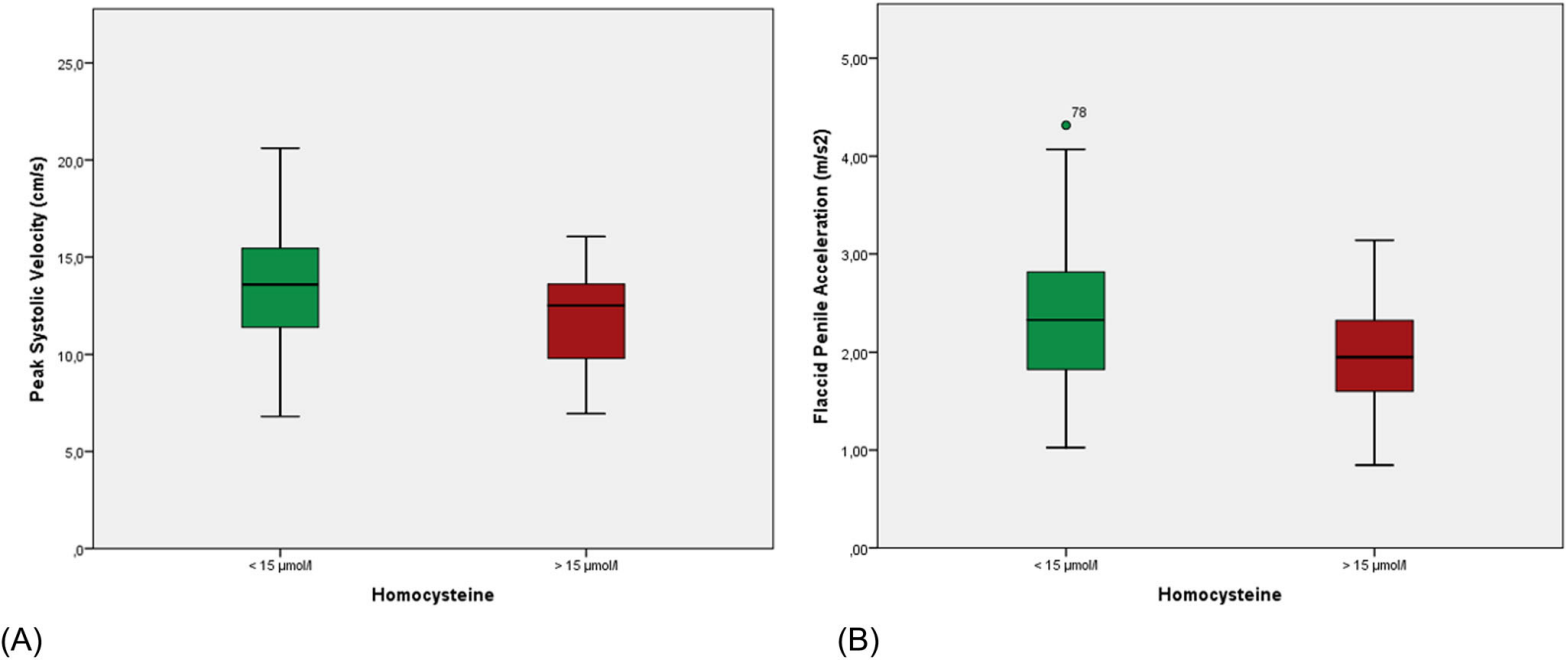
Distribution of values of (A) peak systolic velocity (PSV) and (B) flaccid penile acceleration (FPA) between patients with normal and high levels of homocysteine (Hcys)

**FIGURE 2:**
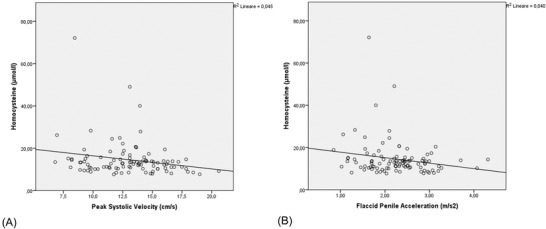
Correlation between homocysteine (Hcys) levels and (A) peak systolic velocity (PSV) and (B) flaccid penile acceleration (FPA)

Clinical and laboratory data of the included patients are listed in Table 1. Mean age of the subjects was 52.1 ± 12.6 years (range: 25–53 years) and mean body mass index (BMI) was 25.6 ± 4.0 kg/m^2^. Mean serum Hcys level was 14.9 ± 9.5 μmol/l, and HHcys was present in 26% patients. Table 2 shows data from B‐PDU. Mean PSV and FPA were 13.1 ± 2.9 cm/s and 2.28 ± 0.70 m/s^2^, respectively. These parameters showed a strong correlation (*r* = 0.690; *p* < 0.001). According to PSV, 50 patients (39.7%) showed arterial insufficiency. Moreover, a pathologic value of FPA was present in six patients (4.8%). Subdividing the patients according to the presence of HHcys, values of both PSV and FPA was significantly lower in subjects with Hcys > 15 μmol/L (Table 3, Figure 1A,B). Post hoc analysis showed 86.7% and 80% statistical power for PSV and FPA, respectively. Moreover, an inverse correlation was present between Hcys and PSV (*r* = ‐0.213; *p* = 0.03) and between Hcys and FPA (*r* = ‐0.199; *p* = 0.05) (Figure 2A,B). In addition, a significant inverse correlation was present between PSV and age (*r* = ‐0.484; *p* < 0.001), BMI (*r* = ‐0.307; *p* = 0.005), glycemia (*r* = ‐0.279; *p* = 0.004), HbA1c (*r* = ‐0.318; *p* = ‐0.003), and triglycerides (*r* = ‐0.288; *p* = 0.007), whereas a significant direct correlation was present between PSV and HDL‐cholesterol (*r* = 0.261; *p* = 0.02) (Table 4). Similarly, we found a significant correlation between FPA and age (*r* = ‐0.445; *p* < 0.001), glycemia (*r* = ‐0.221; *p* = 0.027), HbA1c (*r* = ‐0.251; *p* = ‐0.023), triglycerides (*r* = ‐0.342; *p* = 0.001), and HDL‐cholesterol (*r* = 0.311; *p* = 0.007) (Table 5). On the other hand, Hcys levels showed a significant correlation with age (*r* = 0.287; *p* = 0.003) but not with any other metabolic parameter. Four men were frankly hypogonadal (total testosterone <2.30 ng/ml) and 16 had mild hypogonadism (total testosterone 2.3–3.2 ng/ml). No significant correlations were found between hormone levels and Hcys levels or B‐PDU parameters. Multivariate logistic regression analysis for the relation between Hcys levels and pathologic PSV (dependent variables) after adjustment for age, BMI, glycemia, HbA1c, HDL‐cholesterol, and triglycerides (independent variables) is shown in Table 6 and was not significant.

**TABLE 1:** Clinical and laboratory data of the included patients

Age (years)	52.1 ± 12.6
BMI (kg/m^2^)	25.6 ± 4.0
SBS (mmHg)	123.6 ± 10.7
DBS (mmHg)	80.2 ± 6.9
FSH (mIU/ml)	7.2 ± 8.1
LH (mIU/ml)	4.5 ± 3.0
Prolactin (ng/ml)	6.4 ± 2.8
SHBG (nmol/l)	50.2 ± 24.7
Total testosterone (ng/ml)	4.86 ± 2.10
Glycemia (mg/dl)	96.6 ± 18.4
Insulin (μIU/ml)	10.7 ± 5.8
HOMA‐IR	2.44 ± 1.38
HbA1c (mmol/mol)	39.5 ± 8.6
Total cholesterol (mg/dl)	193.6 ± 38.2
HDL‐cholesterol (mg/dl)	52.7 ± 11.7
LDL‐cholesterol (mg/dl)	120.1 ± 27.5
Triglycerides (mg/dl)	117.3 ± 66.4
PSA (ng/ml)	1.5 ± 1.3
Hcys (μmol/l)	14.9 ± 9.5

*Note*: Data are expressed as mean ± SD.

Abbreviations: BMI, body mass index; DBS, diastolic blood pressure; FSH, follicle‐stimulating hormone; HbA1c, glycosylated hemoglobin; Hcys, homocysteine; HDL, high‐density lipoprotein; HOMA‐IR, homeostatic model assessment—insulin resistance; LDL, low‐density lipoprotein; LH, luteinizing hormone; PSA, prostate specific antigen; SBS, systolic blood pressure; SHBG, sexual hormone‐binding globulin.

**TABLE 2:** Instrumental data (basal penile duplex ultrasound) of the included patients

PSV (cm/s)	13.1 ± 2.9
FPA (m/s^2^)	2.28 ± 0.7
Abnormal PSV (<13 cm/s)	50 (39.7%)
Abnormal FPA (<1.17 m/s^2^)	6 (4.8%)

*Note*: Data are expressed as mean ± SD or *N* (%).

Abbreviations: FPA, flaccid penile acceleration; PSV, peak systolic velocity.

**TABLE 3:** Instrumental data (basal penile duplex ultrasound) of patients with normal versus high levels of homocysteine (Hcys)

	Hcys < 15 μmol/l	Hcys > 15 μmol/l	*p*
PSV (cm/s)	13.4 ± 3.0	11.9 ± 2.3	0.027
FPA (m/s^2^)	2.32 ± 0.65	2.00 ± 0.60	0.034

*Note*: Data are expressed as mean ± SD.

Abbreviations: FPA, flaccid penile acceleration; PSV, peak systolic velocity.

**TABLE 4:** Correlation coefficients of peak systolic velocity (PSV) values with clinical parameters

	*r*	*p*
Age	−0.484	<0.001
BMI	−0.307	0.005
Glycemia	−0.279	0.004
HbA1c	−0.318	0.003
Triglycerides	−0.288	0.007
HDL−cholesterol	0.261	0.02

Abbreviations: BMI, body mass index; HbA1c, glycosylated hemoglobin; HDL, high‐density lipoprotein.

**TABLE 5:** Correlation coefficients of flaccid penile acceleration (FPA) values with clinical parameters

	*r*	*p*
Age	−0.445	<0.001
Glycemia	−0.221	0.027
HbA1c	−0.251	0.023
Triglycerides	−0.342	0.001
HDL−cholesterol	0.311	0.007

Abbreviations: HbA1c, glycosylated hemoglobin; HDL, high‐density lipoprotein.

**TABLE 6:** Multivariate logistic regression analysis for the relation between homocysteine (Hcys) levels and pathologic peak systolic velocity (PSV) (dependent variables) after adjustment for age, body mass index (BMI), glycemia, glycosylated hemoglobin (HbA1c), high‐density lipoprotein (HDL) cholesterol, and triglycerides (independent variables)

	Odds ratio	95% CI	*p*
Age	1.128	1.026–1.240	0.013
BMI	1.094	0.874–1.369	0.434
Glycemia	0.940	0.851–1.039	0.229
HbA1c	1.210	0.920–1.592	0.173
Triglycerides	1.055	0.963–1.157	0.250
HDL‐cholesterol	1.014	0.995−1.033	0.140
Hcys	0.992	0.911−1.081	0.863

Abbreviation: CI, confidence interval.

## 4 DISCUSSION

Emerging evidence suggests a significant association between HHcys and ED. In a recent study based on a wide population of 1318 subjects in which serum levels of Hcys, FA, and vitamin B12 were evaluated, Chen et al.^19^ found out that HHcys is associated to a threefold increased risk of ED, especially for patients >60 years. This was already suggested by Sansone et al.,^9^ who recently conducted a large meta‐analysis on nine different studies, showing that ED patients present higher levels of Hcys rather than control, with a standard mean difference of 1.00 (95% CI 0.65–1.35, *p* < 0.0001). Moreover, Giovannone et al.^20^ have found that plasma levels could potentially be monitored as an early marker of ED prediction and could be used to identify those patients who may go on to develop severe ED.

The results of our cross‐sectional study appear to be in line with the literature, confirming that HHcys is frequently detected in ED patients, being present in about a quarter of our population. Interestingly, we found an inverse correlation between Hcys levels and B‐PDU parameters, namely PSV and FPA. According to “the artery size hypothesis” by Montorsi et al.,^21^ ED represents an early manifestation of atherosclerosis and may precede other cardiovascular complication such as coronary disease. On this purpose, an FPA value <1.17 m/s^2^ has shown a threefold increase in incidence of major adverse cardiovascular events and it has been proposed as a non‐invasive parameter to identify subjects with adverse cardiovascular profiles among ED patients with apparently lower risk.^15^ In our study, only six patients (4.8%) had an FPA <1.17 m/s^2^ but this parameter strongly correlated with PSV. Moreover, a significant inverse correlation between FPA and Hcys levels emerged. This interesting result reflects the previous findings suggesting that HHcys could play a role in the development of atherosclerosis and CVD, as already suggested in the late 1960s and recently confirmed.^22^ Indeed, the role of HHcys in the development of endothelial dysfunction and, consequently, of atherosclerosis, is becoming increasingly evident. In vitro, Hcys impairs the dilatory effect of NO reacting with O_2_^–^ to form peroxynitrite (ONOO^–^) and reducing its bioavailability.^23^ In addition, Hcys increases endothelin‐1 expression, which acts as a potent vasoconstrictor.^5^ Taken together, these actions result in the inability of the endothelium to regulate vascular tone. Lastly, HHcys causes an increase in production and release of reactive oxygen species (ROS), a family of molecules which damages the integrity of the endothelium favoring the development of atherosclerosis.^5^ Anyway, besides the indirect effect on atherosclerosis, HHcys can also impair erectile function with several direct mechanisms. Indeed, HHcys is able to inhibit acetylcholine‐induced relaxation and cGMP production in the rabbit corpus cavernosum in vitro,^23^ whereas, in vivo, it decreases the local production of NO in cavernous sinus and vessels affecting the number and latency of erections after apomorphine injection.^24^ In addition, a decrease of smooth muscle cells and an increase in the fibrosis degree in the corpora cavernosa has been recently observed in a rat model of HHcys.^25^

From a clinical point of view, the association between HHcys and ED has been reported by several authors.^9^, ^19^, ^26^, ^27^ Interestingly, Sansone et al.^9^ reported a higher frequency of HHcys in ED subjects without diabetes mellitus rather than diabetic subjects with ED. Even if it could seem counter‐intuitive, it has been suggested that in the present of significant comorbidities such as diabetes, HHcys could act in a synergistic way and smaller changes in serum Hcys could become clinically relevant. In our study, a significant correlation between Hcys and B‐PDU parameters was present. Moreover, the latter showed a significant correlation with several metabolic parameters such as BMI, fasting glycemia, LDL‐ and HDL‐cholesterol, and triglycerides. Interestingly, Hcys levels did not correlate with any other metabolic parameter, but a significant correlation with age emerged. HHcys is common among elderly people and it is often because of a low intake of FA and B vitamins.^28^ Since FA acts as a cofactor in the re‐methylation process of Hcys,^29^ its role in the development of HHcys‐related ED cannot be excluded. On this purpose, Attia et al.^4^ found an inverse correlation between FA levels and ED severity and that a cut‐off value of 9.42 ng/ml is able to detect ED patients with a sensitivity of 80.0%, a specificity of 93.3%, and an area under curve (AUC) of 0.913. Sansone et al.^9^ reported higher levels of both Hcys and FA in ED patients compared with non‐ED patients, even if, surprisingly, FA levels were not correlated to Hcys levels. In this regard, another important cause of HHcys is MTHFR mutation. The most common MTHFR mutations are C677T and A1298C. In North America, Europe, and Australia, 8%–20% of the population is homozygous for MTHFR C677T mutation, which leads to reduced enzyme function of about 30% of normal (65% for heterozygous individuals), and 7%–12% of the population is homozygous for MTHFR A1298C mutation, which leads to 60% of normal enzyme function.^30^ This could be crucial for younger patients. Indeed, young adult individuals (mean age 32.2 years) with the MTHFR 677TT genotype and the 677TT + 1298AC combined genotype were found to have a 3.16‐ and 3.89‐fold increased risk of developing ED compared with age‐matched healthy controls.^10^

Unfortunately, even if the relationship between HHcys and ED is becoming evident, intervention studies aimed at reducing Hcys levels have yielded inconclusive results. According with the results of a recent Cochrane review, which investigated the effect of B‐complex vitamin (cyanocobalamin or B12, FA, and pyridoxine or B6) administration as Hcys‐lowering intervention, data from 15 randomized controlled trials involving 71,422 participants showed a reduction in the risk of stroke, but no significant effects on the incidence of either myocardial infarction or death from any cause. Based on these results, the review concluded that there is still the need for large cooperative trials in order to compare types of Hcys‐lowering intervention so as to identify the optimal dosage and possible association with other medications.^8^ In this regards, Hcys‐lowering agents could be associated to the standard treatment options for ED such as PDE5 inhibitors (PED5i), testosterone replacement treatment, intracavernosal injection therapy, vacuum constriction devices, intraurethral prostaglandin suppositories, and surgical placement of a penile prosthesis.^1^ Indeed, the combined administration of sildenafil, FA, and vitamin B6 in patients with HHcys because of MTHFR mutation led to a significant reduction in Hcys levels (*p* < 0.001) and to a marked increase in the PDE5i response rate.^31^ Moreover, Hamidi Madani et al.^32^ recently investigated the effect of combined PDE5i and FA on 83 patients with ED who were divided into two groups to receive a combination of tadalafil (10 mg every other day) and FA (5 mg daily) or tadalafil and placebo for 3 months. Even if both groups showed a significant improvement in sexual function after treatment, in the FA group the post‐treatment IIEF score was significantly higher than in the placebo group (16.80 vs. 14.37, *p* = 0.002). Interestingly, Elshahid et al.^29^ recently reported a significant improvement in erectile function (median IIEF‐5 score increased from 6 to 14, *p* < 0.001) and D‐PDU (median PSV increased from 21.45 to 27.6, *p* < 0.001) after FA administration alone (500 mcg/day) for 3 months. Taken together, these data suggest that patients with ED and HHcys could benefit from normalization of Hcys levels, but further studies are needed to confirm these findings.

The strength of our study is that we used for the first time the parameters of B‐PDU, an inexpensive, non‐invasive, and easy‐to‐perform examination, to study the impact of HHcys on male sexual function. To the best of our knowledge, no other author has yet reported similar data on such a numerous group of subjects. Given the role of HHcys in the development of ED and, above all, their relationship with CVD, it seems essential to associate laboratory assessment with instrumental examination in identifying subjects at high cardiovascular risk. On the other hand, we are aware of the limitations of our work first. The cross‐sectional nature of our study and the lack of an intervention to reduce Hcys levels requires further confirmation, ideally by randomized controlled trials. In addition, the assessment of FA levels and the MTHFR genotyping could be useful to identify the source of HHcys and to investigate whether the effectiveness of Hcys‐lowering interventions may be affected by this.

## 5 CONCLUSION

A significant association between hyperhomocysteinemia and velocimetric parameters assessed by basal penile duplex ultrasound was detected in the present study. Other metabolic parameters significantly correlated with both peak systolic velocity and flaccid penile acceleration, but not with homocysteine. Patients with arteriogenic erectile dysfunction showed higher homocysteine levels than patients with peak systolic velocity >13 cm/s. Hyperhomocysteinemia could therefore represent an independent risk factor for erectile dysfunction and, consequently, cardiovascular disease, but further studies are needed to investigate if normalization of homocysteine levels could improve erectile function and prevent forthcoming cardiovascular events.

## CONFLICT OF INTEREST

All authors declare that they have no conflict of interests.

## FUNDING INFORMATION

This study did not receive funds.

## AUTHOR CONTRIBUTIONS

Giancarlo Balercia designed the work and provided overall supervision of the project. Gianmaria Salvio, Melissa Cutini, Lara Giovannini, Michele Perrone, and Alessandro Ciarloni conducted medical visits. Gianmaria Salvio performed US examinations. Simone Cordoni, Nicola Delli Muti, Federica Finocchi, and Francesca Firmani collected the data. Gianmaria Salvio wrote the manuscript and performed the statistical analysis. All authors read and approved the manuscript.
